# Contribution of *PNPLA3*, *GCKR*, *MBOAT7*, *NCAN*, and *TM6SF2* Genetic Variants to Hepatocellular Carcinoma Development in Mexican Patients

**DOI:** 10.3390/ijms26157409

**Published:** 2025-08-01

**Authors:** Alejandro Arreola Cruz, Juan Carlos Navarro Hernández, Laura Estela Cisneros Garza, Antonio Miranda Duarte, Viviana Leticia Mata Tijerina, Magda Elizabeth Hernández Garcia, Katia Peñuelas-Urquides, Laura Adiene González-Escalante, Mario Bermúdez de León, Beatriz Silva Ramirez

**Affiliations:** 1 Department of Gastroenterology and Hepatology, High Specialty Medical Unit 25, Mexican Social Security Institute, Monterrey 64320, NL, Mexico; alejandro.arreola@imss.gob.mx (A.A.C.); dr.juancarlosnavarrohernandez@gmail.com (J.C.N.H.); hepatologia@dralauracisneros.com (L.E.C.G.); 2 Department of Genomic Medicine, Instituto Nacional de Rehabilitación “Luis Guillermo Ibarra Ibarra”, Mexico City 14389, Mexico; amiranda@inr.gob.mx; 3 Department of Immunogenetics, Northeast Biomedical Research Centre, Mexican Social Security Institute, Monterrey 64320, NL, Mexico; viviana.mata@imss.gob.mx; 4 Department of Parasitology and Pharmacognosy, Northeast Biomedical Research Centre, Mexican Social Security Institute, Monterrey 64320, NL, Mexico; magda.hernandezg@imss.gob.mx; 5 Department of Molecular Microbiology, Northeast Biomedical Research Centre, Mexican Social Security Institute, Monterrey 64320, NL, Mexico; katia.penuelasu@imss.gob.mx (K.P.-U.); laura.gonzaleze@imss.gob.mx (L.A.G.-E.); 6 Department of Molecular Biology, Northeast Biomedical Research Centre, Mexican Social Security Institute, Monterrey 64320, NL, Mexico; mario.bermudez@imss.gob.mx

**Keywords:** hepatocellular carcinoma, single nucleotide polymorphisms, risk factors, genetic variants

## Abstract

Hepatocellular carcinoma (HCC) is the most prevalent subtype of liver cancer with an increasing incidence worldwide. Single nucleotide polymorphisms (SNPs) may influence disease risk and serve as predictive markers. This study aimed to evaluate the association of *PNPLA3* (rs738409 and rs2294918), *GCKR* (rs780094), *MBOAT7* (rs641738), *NCAN* (rs2228603), and *TM6SF2* (rs58542926) SNPs with the risk of developing HCC in a Mexican population. A case-control study was conducted in unrelated Mexican individuals. Cases were 173 adults with biopsy-confirmed HCC and 346 were healthy controls. Genotyping was performed using TaqMan allelic discrimination assay. Logistic regression was applied to evaluate associations under codominant, dominant, and recessive inheritance models. *p*-values were corrected using the Bonferroni test (pC). Haplotype and gene–gene interaction were also analyzed. The GG homozygous of rs738409 and rs2294918 of *PNPLA3*, TT, and TC genotypes of *GCKR*, as well as the TT genotype of *MBOAT7*, were associated with a significant increased risk to HCC under different inheritance models (~Two folds in all cases). The genotypes of *NCAN* and *TM6SF2* did not show differences. The haplotype G-G of rs738409 and rs2294918 of *PNPLA3* was associated with an increased risk of HCC [OR (95% CI) = 2.2 (1.7–2.9)]. There was a significant gene–gene interaction between *PNPLA3* (rs738409), *GCKR* (rs780094), and *MBOAT7* (rs641738) (Cross-validation consistency (CVC): 10/10; Testing accuracy = 0.6084). This study demonstrates for the first time that *PNPLA3* (rs738409 and rs2294918), *GCKR* (rs780094), and *MBOAT7* (rs641738) are associated with an increased risk of developing HCC from multiple etiologies in Mexican patients.

## 1. Introduction

Hepatocellular carcinoma (HCC) is the predominant subtype of liver cancer. In 2023, approximately one million new cases were diagnosed, and more than 800,000 deaths were attributed to this kind of cancer, making it one of the leading causes of cancer-related deaths worldwide [1]. Its incidence is expected to grow over the next two decades; therefore, immediate interventions are necessary for its timely detection, and management [2].

Several factors contribute to chronic damage in the liver, which increases the risk for the development of HCC [3]. The most common etiologies include chronic viral infection with hepatitis B virus (HBV) or hepatitis C virus (HCV), excessive alcohol intake (ALD), aflatoxin B1 (AFB1) contaminated food, and rare monogenic diseases such as hemochromatosis or alpha-1-antitrypsin deficiency [4,5]. Over the past few years, HBV and HCV-related HCC cases have decreased due to universal HBV vaccination and effective antiviral treatments [5]. However, non-alcoholic fatty liver disease (NAFLD) and non-alcoholic steatohepatitis (NASH) associated with metabolic disorders such as obesity, insulin resistance, and type 2 diabetes (T2DM) have emerged as the fastest-growing risk factors, and they are predicted to become the leading cause of HCC. Genetic background, male sex, older age, and environmental factors also influence the development and progression of HCC [6,7].

Genetic studies based on family aggregation and twin studies have described genetic factors that contribute to susceptibility and an inter-individual variability of disease [8,9]. Genome-wide association studies (GWAS) have led to the identification of genetic variants that modify an individual’s predisposition to liver disease progression from fibrosis to cirrhosis, and HCC. In this context, rs738409 (C > G), a single nucleotide polymorphism (SNP) in the Patatin-like phospholipase domain containing 3 (*PNPLA3*) gene, has been strongly associated with the full spectrum of liver disease [10,11,12,13]. This non-synonymous variant is characterized by an isoleucine-to-methionine substitution at position 148 (I148M). The wild type of protein exhibits activity as triglyceride lipase, while the I148M variant is resistant to normal proteasomal degradation and accumulates in hepatic lipid droplets. Consequently, lipotoxicity and oxidative DNA damage induce hepatocarcinogenesis [14]. The rs2294918, another SNP in *PNPLA3*, is associated with reduced expression of the PNPLA3 protein, lowering the effect of the rs738409: G variant on predisposition to steatosis and liver damage [15].

Other SNPs of genes related to altered hepatic lipid metabolism have also been associated with HCC, such as: rs58542926 (p.Glu167Lys) of the Transmembrane 6 superfamily member 2 (*TM6SF2*) [16], rs780094 (intronic variant) of Glucokinase regulator (*GCKR*) [17,18], rs2228603 (p.Pro92Ser) of Neurocan *(NCAN*) [19], and rs641738 (p.Glu17Val) of Membrane bound O-acyltransferase domain containing 7 (*MBOAT7*) [20].

Currently, in Mexico, the epidemiological transition due to the aging of its population, along with a high prevalence of metabolic syndrome, type 2 diabetes mellitus, and obesity, has led to a considerable increase in the incidence of HCC. The prevalence in Mexico is moderate compared to some Asian countries and is comparable to certain regions in Europe and the United States. The variations in prevalence across these regions can be attributed to differences in risk factors, access to healthcare, and public health initiatives aimed at preventing liver disease [1,2].

The identification of genetic variants that contribute to the predisposition to HCC could facilitate the prevention and early diagnosis of the disease, thus establishing individualized therapy and better clinical outcomes. The aim of the present study was to analyze the genetic association, as well as the interaction, of SNPs of *PNPLA3* (rs738409 and rs2294918), *GCKR* (rs780094), *MBOAT7* (rs641738), *NCAN* (rs2228603), and *TM6SF2* (rs58542926) with the risk of development of HCC in a Mexican population.

## 2. Results

### 2.1. Subjects Characteristic

A total of 519 subjects were enrolled in the study, including 173 HCC cases and 346 controls. There were no significant differences in age or sex between the groups (*p* > 0.05). The demographic and clinical characteristics of the patients and controls are depicted in (Table 1). The body mass index (BMI) was not normally distributed and showed medians of 28.42 ± 4.01 and 25.7 ± 3.4 kg/m^2^ in patients and controls, respectively (*p* = 0.08). The frequency of comorbidities, including type 2 diabetes and hypertension, was significantly higher in HCC patients than in controls (*p* < 0.0001 and *p* = 0.04, respectively). The BCLC staging of patients with HCC revealed that 55.4% were in the earlier stages (A and B), while 44.6% were classified as having advanced or end-stage disease (C and D). The Child–Pugh score indicated that 50% of HCC cases were predominantly classified as A, 37.8% as B, and 12.2% as C. The most common etiology of HCC was ALD (38%), followed by NAFLD (31%), HCV infection (17%), autoimmune liver disease (5%), and unknown cause (9%).

### 2.2. Analysis of the Association Between SNPs and Cases-Controls

Allele frequencies for all SNPs in the control group were consistent with Hardy–Weinberg equilibrium (*p* > 0.05). The allelic distribution of rs2228603 of *NCAN* and rs58542926 of *TM6SF2* were similar in patients with HCC and the control group. Conversely, the allelic frequencies of rs738409 and rs2294918 of *PNPLA3,* rs780094 of *GCKR,* and rs641738 of *MBOAT7* demonstrated significant differences between HCC patients and controls. Regarding the SNPs of PNPLA3, the G allele of rs738409 was more frequent in patients than in controls (73% vs. 56%, OR (95% CI) = 2.2 (1.6–2.9); Pc < 0.0001). As well as the G allele of rs2294918 (86% vs. 80%, OR (95% CI) = 1.6 (1.1–2.3); *p* = 0.01). Similarly, the T allele of rs780094 of *GCKR* (38% vs. 29%, OR (95% CI) = 1.4 (1.1–1.9); Pc = 0.007), and T allele of rs641738 of *MBOAT* (44% vs. 33%, OR (95% CI) = 1.5 (1.1–2.0); Pc = 0.001) were more frequent in cases. The allelic frequencies of both groups are shown in (Table 2).

### 2.3. The Inheritance Model Analysis of Polymorphisms in Patients with HCC and Controls

Table 3 outlines the genotype frequencies of the SNPs studied between HCC patients and controls. In regard to SNPs of *PNPLA3*, the homozygous genotype GG of rs738409 was associated with high risk of developing HCC under codominant model (OR (95% CI) = 4.5 (2.3–8.9), *p* < 0.0001), dominant model (OR (95% CI) = 2.8 (1.5–5.5), *p* = 0.001), and recessive model (OR (95% CI) = 2.7 (1.8–4.1), *p* < 0.0001). Similarly, the homozygous genotype GG of rs2294918 was associated with an increased risk of HCC under a recessive model (OR (95% CI) = 1.8 (1.1–2.7), *p* < 0.01). Likewise, the homozygous genotype TT of rs780094 of *GCKR* was associated with an increased risk of developing HCC under a codominant model (OR (95% CI) = 2.2 (1.2–3.9), *p* = 0.009), dominant model (OR (95% CI) = 1.8 (1.2–2.7), *p* = 0.003), and a recessive model (OR (95% CI) = 1.7 (0.9–3.0), *p* = 0.05). Regarding the heterozygote genotype TC, it was found to be associated under a codominant model (OR (95% CI) = 1.7 (1.1–2.6), *p* = 0.01). Finally, under codominant, dominant, and recessive models, the TT genotype of rs641738 of *MBOAT7* was associated with a significantly increased risk of HCC (OR (95% CI) = 2.9 (1.6–5.1), *p* = 0.001; 1.7 (1.1–2.7), *p* = 0.001; and 2.2 (1.3–3.6), *p* = 0.003, respectively). TC heterozygote genotype of rs641738 only showed an increased risk for HCC under a codominant model (OR (95% CI) = 1.7 (1.1–2.7); *p* = 0.02). The genotypes of SNPs of *NCAN* and *TM6SF2* did not show significant differences between HCC patients and controls.

### 2.4. Analysis of PNPLA3 Haplotypes in Cases and Controls

Haplotypes of the SNPs of *PNPLA3,* rs738409, and rs2294918; were constructed and their association with HCC was also analyzed. The GG haplotype was significantly associated with an increased risk of HCC (OR (95% CI) = 2.2 (1.7–2.9), *p* < 0.0001) that was not previously documented. Conversely, the CG haplotype appeared to confer a protective effect against HCC, with an OR (95% CI) of 0.5 (0.3–0.7) (*p* <0.0001) (Table 4).

On the other hand, homozygous genotype GG of rs738409 was associated with HCC patients of different etiologies (NAFLD = 53, ALD = 65, HCV = 30), (all OR (95% CI) = 2.68 (1.4–5.0), *p* = 0.003). The same trend of association was found in HCC patients classified by BCLC (A = 44, B = 52, C = 48, D = 29) (all OR (95% CI) = 2.68 (1.4–5.0), *p* < 0.003), and Child–Pugh (A = 82, B = 62, C = 20), (all OR (95% CI) = 3.07 (1.8–5.1), *p* < 0.0001) (Supplementary Table S1).

### 2.5. Gene-Gene Interaction Analysis

In the gene–gene interaction analysis, the MDR algorithm yielded three models; nevertheless, the model that showed the most consistent results was that formed by *PNPLA3* (rs738409), *GCKR* (rs780094), and *MBOAT7* (rs641738) with a CVC of 10/10 and a Testing accuracy of 0.6084 (*p* = 0.001), demonstrating an epistatic effect of these genes on HCC risk in the Mexican population. Additionally, that model was associated with an increased risk to HCC (Table 5).

## 3. Discussion

Disparities in the distribution of genetic and environmental risk factors among ethnic groups are unquestionably linked to the incidence of HCC. The availability of prognostic markers with high sensitivity and specificity is crucial for early diagnosis, which potentially reduces the incidence and mortality of HCC where their effective implementation is essential in clinical practice.

The purpose of this case-control study was to examine associations between the selected genetic variants in Mexican patients and biopsy-confirmed HCC. We demonstrated for the first time that SNPs of *PNPLA3* (rs738409 and rs2294918), *GCKR* (rs780094), and *MBOAT7* (rs641738) are associated with HCC in Mexican patients. Nevertheless, we found no association of *NCAN* (rs2228603) and *TM6SF2* (rs58542926) with HCC, which had previously been identified in other populations. Knowing the genetic predisposition of populations to HCC is a valuable insight for identifying preventive and therapeutic strategies.

The genetic variants analyzed in this study belong to genes that affect lipid metabolism, which could have a cumulative effect on the development of HCC. The rs738409G (I148M) of *PNPLA3* is a genetic variant that has been consistently linked to elevated liver enzymes, hepatic triglyceride accumulation (steatosis), and more aggressive disease manifestations, including non-alcoholic steatohepatitis, advanced fibrosis, cirrhosis, and even hepatocellular carcinoma with different etiologies [21,22]. In 2008, Romeo et al. [10] performed a genome-wide association analysis for the first time in a multiethnic population, revealing a strong association between rs738409 (I148M) and the risk of NAFLD development. Intriguingly, the frequency of this variant was higher in the Hispanic population and lower in European Americans and African Americans. Liu et al. [12], in 2014, recruited a large European cohort confirming the association between rs738409G (I148M) genotype and NAFLD-related HCC, independently of potentially confounding factors including age, gender, co-existent diabetes, obesity, and the presence of cirrhosis. Since then, different studies have shown that the I148 M variant has a higher risk of HCC in individuals with alcoholic liver disease [23,24], NAFLD [25,26], and viral hepatitis [27,28]. Our study is in line with those previous reports, as our findings suggest that the G allele of the variant rs738409 is significantly associated with HCC in the Mexican population.

Similarly, PNPLA3 1148M polymorphism was likewise associated with early and advanced stages of Child–Pugh and BCLC scores. The published literature is consistent with our data [13,24,25,26,27,28]. Prospective cohorts of patients with early-stage HCC need to be evaluated to assess the usefulness of the I148M polymorphism as a staging marker for HCC.

PNPLA3 protein displays lipase activity for triglycerides and retinol esters and acyltransferase activity for phospholipids in hepatocytes and hepatic stellate cells, respectively. Genetic variant rs738409 (I148M), produces a loss of function leading to the accumulation of triglycerides in hepatocytes [29,30]. Accordingly, this is resistant to normal proteasomal degradation [14,31], resulting in the accumulation of hepatic lipid droplets and gradual liver injury, hepatocellular degeneration, and cancer development. Comparably, the 1148M variation in hepatic stellate cells loses its hydrolase activity, which results in extracellular matrix deposition and intracellular retinol retention, causing cell activation. This activation leads to profibrosis and exacerbation of the inflammatory response, which ultimately leads to severe liver fibrosis [32]. Recent research has examined the connection between the I148M variant and rs2294918 of *PNPLA3*. The simultaneous existence of these two SNPs reduces liver damage and lessens the effect of I148M on the accumulation of lipid droplets [15].

The presence of a non-synonymous variant of *TM6SF2* (rs58542926) results in a misfolded protein, with accelerated degradation and reduced function, leading to retention of low-density lipoproteins (VLDL), promoting hepatic steatosis, inflammation, and fibrosis, accelerating the development of HCC. Patients with this variant have a 1.92-fold greater risk of developing HCC in comparison to patients without this variant [16]. *MBOAT* (Glu17Val, rs641738) is the other genetic variant that has been associated with hepatocellular fat accumulation and the production of inflammatory mediators. Both hepatic remodeling composition and plasma phosphatidylinositol are altered by the rs641738 T allele. Patients who carry this variant are 2.1 times more likely to develop HCC than patients who do not carry it [20]. The intronic variant *GCKR* rs780094 is associated with a variety of lipid metabolism disorders that increase fat production by inducing glycolysis [17]. Similarly, the altered levels of VLDL and triglycerides were associated with the *NCAN* gene SNP rs2228603, which is expressed in both liver and neural tissue. *NCAN* may be involved in transport processes within the liver. Hepatic lipid accumulation could promote carcinogenesis. Recent studies have identified *NCAN* rs2228603 in individuals of European ancestry but not in the Asian population [19].

The *TM6SF2* rs58542926 and *NCAN* rs2228603 variants were not associated with hepatocellular carcinoma in our study population. Their frequency was less common in the Mexican population, which might hide their influence on liver disease, given the well-established role of *TM6SF2* and *NCAN* variants in liver disease (Supplementary Table S2).

Several reports on Mexican populations by Larrieta-Carrasco et al. [33,34], Flores et al. [35], Martínez et al. [36], and Chinchilla-López et al. [37] collectively highlight the significant role of the *PNPLA3* I148M G variant in liver health in several populations, particularly among overweight/obese Mexican children and indigenous/mestizo adults. These investigations reveal a consistent association between this genetic polymorphism and elevated alanine transaminase (ALT) levels, an increased risk of liver diseases such as non-alcoholic fatty liver disease (NAFLD). Likewise, homozygous GG carriers had a 3.8-fold increased risk of steatohepatitis [37]. The prevalence of the 148 G risk allele is significantly higher in the Mexican mestizo and indigenous population (56%), than reported in other populations [13]. The elevated frequency of the I148M allele is due to its indigenous component and may be considered an important risk factor for liver damage [33,34,35,36,37]. The Mexican population is a result of a unique mixture of the autochthonous inhabitants of the region (38%) with European (59%) and African (3%) immigrants who came to America in the XVI century [38].

Epistasis is the interaction between two or more genes. This can modify the genetic associations and can be a factor in the susceptibility to developing a disease [39,40]. In this study, we analyze epistasis through the MDR algorithm. This approach was designed specifically to detect, characterize, and interpret gene–gene interactions independently of the single effects of the genes [41]. Previously, it has been described that epistasis may be implicated in HCC [40,42,43]. Our results suggest that there is an interaction between *PNPLA3* (rs738409), *GCKR* (rs780094), and *MBOAT7* (rs641738) contributing to HCC development. To our knowledge, this is the first time that interaction between those genes contributing to HCC has been reported.

This study had certain limitations. First, this was a hospital-based case-control study; therefore, there is inevitably a retrospective data bias. The sample size could be a disadvantage. In this regard, achieving a large sample size of hospital-based case-control studies may be a challenge. We also recognize that it was not possible to obtain data on controls such as chronic alcohol consumption, hepatitis B, hepatitis C, and non-alcoholic fatty liver disease, which are recognized as risk factors associated with HCC. Therefore, we are aware that there is a lack of control during the statistical analysis of these potential confounders.

## 4. Material and Methods

### 4.1. Subjects

This case-control study was conducted in compliance with the Helsinki Declaration and was approved by the Research and Ethics Committee of the Mexican Social Security Institute (IMSS-2018-785-050). All subjects gave written informed consent.

A total of 519 unrelated Mexicans were consecutively recruited, between October 2018 and September 2021, from the Gastroenterology Department at the High Specialty Medical Unit 25, and Family Medicine IMSS Clinics from Monterrey, Nuevo Leon, Mexico.

Cases were adult patients with a diagnosis of HCC confirmed by biopsy. Their demographic, laboratory, and clinical data were collected. The viral-related HCC etiology was determined based on positive serology for HCV or HBV (anti-HCV IgG with positive RNA at PCR and Hepatitis B surface antigen (HBsAg), respectively). Autoimmune-related HCCs were determined by antibody tests, including anti-nuclear, anti-actin, or both. Alcohol-related HCC was based on a history of persistent high alcohol intake (>80 g/day in men and >60 g/day in women for at least 10 years). NAFLD-related HCC was established in patients with metabolic syndrome but without both chronic viral infection and high alcohol intake. HCC of unknown cause was defined as the absence of any previously mentioned etiologies. The severity of liver injury was assessed by the Child–Pugh score, and the Barcelona Clinic Liver Cancer (BCLC) staging system for determining prognosis.

**Figure 1:**
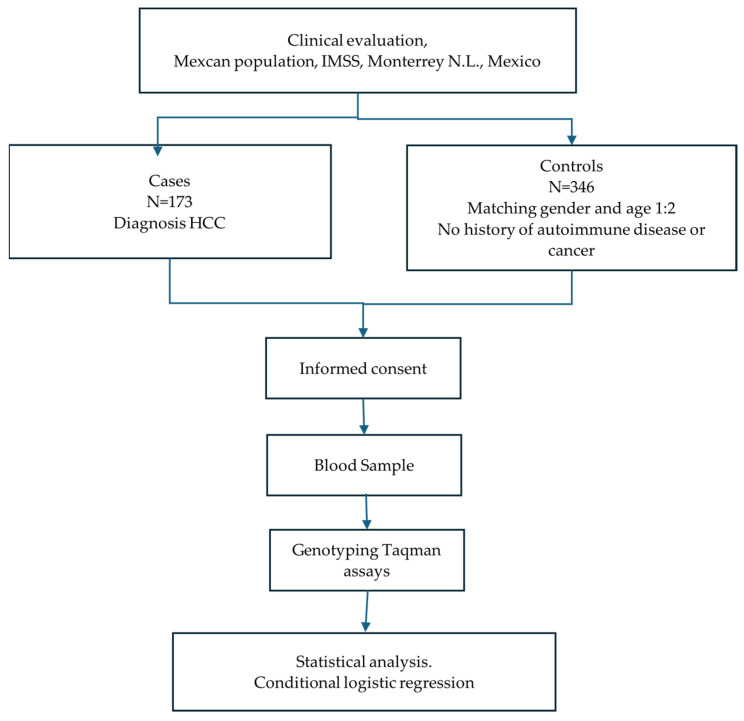
Overview of the study design.

Controls were matched to cases for gender and age. They were ambulatory patients who attended for minor medical problems or as part of preventive medicine programs, with no family history of autoimmune disease or cancer. Additionally, their ALT and AST levels were within normal ranges, indicating no liver dysfunction. Furthermore, there were no reported issues related to alcohol consumption. Participants from both groups were of self-reported Mexican-Mestizo ancestry by at least three generations. The screening flowchart of the subjects is shown in Figure 1.

### 4.2. SNPs Selection and Genotyping

The selection of genetic polymorphisms associated with HCC was based on reports of genome-wide association studies (GWAS), exome, and meta-analyses in different populations. We used the dbSNP database (http://www.ncbi.nlm.nih.gov, accessed on 30 June 2020) and Ensembl Genome Browser (https://grch37.ensembl.org/index.html, accessed on 30 June 2020) to obtain their population distribution data (polymorphic loci with an MAF ≥ 0.05) (Table 6).

Genomic DNA was extracted from peripheral mononuclear cells using Qiagen FlexiGene^®^ DNA Kit (Qiagen, Hilden, Germany, and Catalogue No. 51206). DNA quality of each sample was assessed by NanoDrop 2000 UV-Vis Spectrophotometer (Thermo Fisher Scientific Inc., Waltham, MA, USA). DNA samples were stored at −20 °C until use. The SNPs of PNPLA3 (rs738409 and rs2294918), GCKR (rs780094), MBOAT7 (rs641738), NCAN (rs2228603) and TM6SF27 (rs58542926) were determined with an ABI 7500 Fast Real-Time PCR System (Applied Biosystems, Foster City, CA, USA) using TaqMan^®^ SNP Assays (Thermo Fisher Scientific, Catalogue No. 4351379; Assay ID rs738409: C__7241_10, rs2294918: C__16188704_10, rs780094: C___2862873_10, rs641738: C___8716820_10, rs2228603: C__16171492_10, rs58542926: C__89463510_10). Reactions were conducted according to the manufacturer’s instructions. The PCR consisted of a pre-PCR read at 60 °C for 30 s; holding stage at 95 °C for 10 min; and 50 denaturing cycles at 95 °C for 15 s, annealing/extension at 65 °C for 1 min, and post-PCR read at 60 °C for 30 s. All assays were performed in 5 μL reactions, using TaqMan Genotyping Master-Mix (Thermo Fisher Scientific, Catalogue No. 4318157) on 96-well plates. In each essay, three samples with known genotypes and two negative controls were included. Approximately 10% of samples were randomly selected and duplicated for quality control (the concordance rate between duplicates was 100%). Data analysis was performed using TaqMan Genotyper^®^. Software V2.3.

### 4.3. Statistical Analysis

The continuous and categorical variables were expressed as mean ± standard deviation (SD) and percentages, respectively. The comparisons were performed using Student’s t and Chi-squared (χ^2^) tests. Allelic and genotypic frequencies for each gene variant were calculated in both study groups. Hardy–Weinberg equilibrium (HWE) of all SNPs was evaluated by the Chi-squared (χ^2^) test in normal controls. The associations of the genotypes with HCC were tested under the following inheritance model: codominant, dominant, and recessive. To estimate the association magnitude, unadjusted and adjusted odds ratios (OR) with their 95% confidence intervals (95% CI) were calculated through logistic regression considering each genotype as the main effect. All *p*-values were corrected (pC) by the Bonferroni test. The pC values < 0.05 were considered statistically significant. Statistical analyses were performed using STATA ver. 15.0. Haplotypes of SNPs of PNPLA3 were constructed and their association with HCC was analyzed using Haploview version 4.1 (Broad Institute of MIT and Harvard, Cambridge, MA, USA). To detect gene–gene interaction, the multifactor dimensionality reduction software (MDR 3.0.2) was used. This is available at epistasis.org (accessed on 30 June 2020) and the full procedure of the MDR algorithm can be reviewed elsewhere [41,44]. Briefly, all data are randomly divided into 10 parts. From the pool of genetic factors, a set of n factors is selected, and all possible combinations of factors are evaluated for their ability to classify cases and controls in 9/10 of the data (training set) to select the best n-factor model. The remaining 1/10 data (testing set) is used for independent testing for cross-validation consistency (CVC), which indicates the number of times a particular set of factors is identified in each possible 9/10 of the subjects. The process is repeated 10 times with the data split into 10 different training and testing sets. The best models are yielded with their training and testing accuracy (i.e., the proportion of subjects that were grouped correctly according to their status), the CVC, and the statistical significance of the model (evaluated using MDR Permutation Testing Software). The best model is that with the highest CVC and testing accuracy values since this model shows more consistent results. Additionally, (OR (95% CI)) is provided.

## 5. Conclusions

We demonstrated for the first time that in Mexican patients, PNPLA3 (rs738409 and rs2294918), GCKR (rs780094), and MBOAT7 (rs641738) SNPs are associated with the risk of developing HCC from multiple etiologies. Furthermore, we determined that haplotypes composed of PNPLA3 (rs738409 and rs2294918) GG, were associated with the highest risk of HCC (OR = 2.2, *p* < 0.0001). Nevertheless, we found no association between NCAN (rs2228603) and TM6SF2 (rs58542926) SNPs and the risk of HCC. Finally, based on our results from MDR, PNPLA3 rs738409, GCKR rs780094, and MBOAT7 rs641738, represent the best model associated with increased risk of HCC. The early detection and prevention of hepatocellular carcinoma (HCC) is crucial for improving patient outcomes. The integration of emerging biomarkers, advanced imaging techniques, and clinical risk scores will enhance the accuracy of HCC detection and enable personalized screening strategies. This tailored approach aims to improve the prognosis for high-risk patients through precision prevention methods. These genetic variants may serve as risk markers for the development of hepatocellular carcinoma (HCC) in Mexican patients. However, further studies involving larger cohorts from different regions of Mexico are necessary for their predictive implementation.

## Figures and Tables

**Table 1:** Demographic and clinical characteristics of the study groups.

Clinical Data	Casos *n* = 173	Controles *n* = 346	*p*
Age, y, mean ± SD	63 ± 9.9	62.9 ± 10	0.4
Male sex, *n* (%)	102 (58.96%)	204 (58.96%)	1
Female sex, *n* (%)	71 (41.04%)	142 (41.04%)	1
BMI, kg/m^2^ mean ± SD	28.42 ± 4.01	25.7 ± 3.4	0.08
Type II diabetes mellitus, *n* (%)	95 (55%)	102 (29.41%)	<0.0001
Hypertension, *n* (%)	55 (32%)	80 (23.12%)	0.04
Cirrhosis	157 (90%)		
Child Pugh			
A	82 (50%)		
B	62 (37.8%)		
C	20 (12.2%)		
BCLC			
A	44 (25.4%)		
B	52 (30.0%)		
C	48 (27.8%)		
D	29 (16.8%)		
**Etiology of liver disease, *n* (%)**			
Non- alcoholic fatty liver disease (NAFLD)	53 (31%)		
Alcoho liver disease (ALD)	65 (38%)		
Hepatitis C virus disease (HCV)	30 (17%)		
Hepatitis B virus disease (HBV)			
Autoinmune liver disease	9 (5%)		
Unknown liver disease	16 (9%)		

**Table 2:** Allelic association of genes in HCC.

Gene	SNP	Allele	Cases *n* = 173	Controls *n* = 346	OR (CI 95%)	*p* Value	HWE *p*
*PNPLA3*	rs738409	G	254 (73%)	387 (56%)	2.2 (1.6–2.9)	<0.0001	0.8
		C	92 (27%)	305 (44%)	0.4 (0.3–0.6)		
*PNPLA3*	rs2294918	G	299 (86%)	553 (80%)	1.6 (1.1–2.3)	0.01	0.9
		A	47 (14%)	139 (20%)	0.62 (0.43–0.8)		
*GCKR*	rs780094	T	130 (38%)	203 (29%)	1.4 (1.1–1.9)	0.007	0.07
		C	216 (62%)	489 (71%)	0.6 (0.5–0.9)		
*MBOAT7*	rs641738	T	151 (44%)	231 (33%)	1.5 (1.1–2.0)	0.001	0.12
		C	195 (56%)	461 (67%)	0.6 (0.5–0.8)		
*TM6SF2*	rs58542926	T	18 (5%)	34 (5%)	1.1 (0.5–1.9)	0.8	0.33
		C	328 (95%)	658 (95%)	0.9 (0.5–1.7)		
*NCAN*	rs2228603	C	6 (2%)	12 (2%)	1 (0.37–2.68)	1.0	0.7
		T	340 (98%)	680 (98%)	1 (0.37–2.68)		

HWE: Hardy-Weinberg Equilibrium.

**Table 3:** The inheritance models analysis of polymorphisms in patients with HCC and controls.

Gene	Cases	Controls	OR (CI 95%)	*p*	OR (CI 95%) ^a^	*p* ^a^
*PNPLA3 rs738409*						
Codominant						
GG	95 (55%)	107 (31%)	4.2 (2.2–7.9)	<0.0001	4.5 (2.3–8.9)	<0.0001
CG	64 (37%)	173 (50%)	1.7 (0.9–3.3)	0.09	1.9 (0.9–3.7)	0.07
CC	14 (8%)	66 (19%)	1			
Dominant						
GG/CG vs. CC	159 (91.9%)	280 (80.9%)	2.6 (1.4–4.9)	0.002	2.8 (1.5–5.5)	0.001
Recessive						
GG vs. CG/CC	95 (54.9%)	107 (30.9%)	2.7 (1.9–3.9)	<0.0001	2.7 (1.8–4.1)	<0.0001
*PNPLA3 rs2294918*						
Codominant						
GG	130 (75%)	221 (64%)	2.0 (0.6–6.4)	0.2	2.7 (0.8–9.3)	0.09
AG	39 (23%)	111 (32%)	1.2 (0.4–3.9)	0.7	1.7 (0.5–5.8)	0.4
AA	4 (2%)	14 (4%)	1			
Dominant						
GG/AG vs. AA	332 (95.9%)	169 (97.7%)	1.8 (0.6–5.5)	0.3	2.4 (0.7–8.0)	0.1
Recessive						
GG vs. AG/AA	130 (75.1%)	221 (63.9%)	1.7 (1.1–2.6)	0.01	1.8 (1.1–2.7)	0.01
*GCKR rs780094*						
Codominant						
TT	30 (17%)	37 (11%)	1.9 (1.1–3.4)	0.01	2.2 (1.2–3.9)	0.009
TC	70 (41%)	129 (37%)	1.3 (0.9–2.0)	0.1	1.7 (1.1–2.6)	0.01
CC	73 (42%)	180 (52%)	1		1	
Dominant						
TT/TC vs. CC	100 (58%)	166 (48%)	1.5 (1.01–2.2)	0.03	1.8 (1.2–2.7)	0.003
Recessive						
TT vs. TC vs. CC	30 (17%)	37 (11%)	1.7 (1.04–2.9)	0.03	1.7 (0.9–3.0)	0.05
*MBOAT7 rs641738*						
Codominant						
TT	38 (22%)	45 (13%)	2.2 (1.3–3.9)	0.002	2.9 (1.6–5.1)	0.001
TC	75 (43%)	141 (41%)	1.4 (0.9–2.2)	0.09	1.7 (1.1–2.7)	0.02
CC	60 (35%)	160 (46%)	1			
Dominant						
TT/TC vs. CC	113 (65%)	186 (54%)	1.6 (1.1–2.4)	0.01	1.7 (1.1–2.7)	0.001
Recessive						
TT vs. TC/CC	38 (22%)	45 (13%)	1.9 (1.1–3.1)	0.009	2.2 (1.3–3.6)	0.003
*TM6SF2 rs58542926*						
Codominant						
TT	1 (0.6%)	0 (0%)				
TC	16 (9%)	34 (10%)	0.9 (0.5–1.8)	0.8	1.02 (0.5–1.9)	0.9
CC	156 (90%)	312 (90%)	1		1	
Dominant						
TT/TC vs. CC	17 (9.8)	34 (9.8)	1.0 (0.5–1.9)	1.0	1.1 (0.5–2.1)	0.8
Recessive						
TT vs. TC/CC	1 (0.6%)	0 (0%)		0.1		
*NCAN rs2228603*						
Codominant						
CC	167 (97%)	334 (97%)	1			
TC	6 (3%)	12 (3%)	1 (0.3–2.9)	1	1.3 (0.4–4.0)	0.6
TT	0	0				
Dominant						
CC/TC vs. TT	6 (3%)	12 (3%)	1 (0.3–2.9)	1	1.3 (0.4–4.0)	
Recessive						
TT vs. TC/CC	0 (0.0%)	0 (0.0%)				

^a^: adjusted by age and sex.

**Table 4:** Haplotypes analysis between *PNPLA3* rs*738409* and rs*2294918* polymorphisms in the study groups.

Haplotype	Case	Control		
rs738409, rs2294918	HF (%)	HF (%)	OR (CI95%)	*p*
GG	0.723	0.543	2.2 (1.7–2.9)	<0.0001
CG	0.142	0.256	0.5 (0.3–0.7)	<0.0001
CA	0.124	0.184	0.6 (0.4–0.9)	0.0139
GA	0.012	0.016	0.6 (0.2–1.8)	0.5318

Abbreviations: HF, Haplotype frequency; *p*: *p* value.

**Table 5:** Multifactor dimensionality reduction interaction model.

Model	Training Accuracy	Testing Accuracy	CVC	*p*	OR (95% CI)
*PNPLA3* rs738409, *GCKR* rs780094	0.6344	0.6055	7/10	0.002	2.9 (2.03–4.3)
*PNPLA3* rs738409, *GCKR* rs780094, *MBOAT7* rs641738	0.6651	0.6084	10/10	0.001	3.9 (2.6–5.8)
*PNPLA3* rs738409, rs2294918, *GCKR* rs780094, *MBOAT7* rs641738	0.6867	0.5839	10/10	0.01	4.7 (3.1–6.9)

CVC: cross-validation consistency. The best model is referred with the highest testing accuracy and CVC values.

**Table 6:** The SNPs analyzed in this study.

Gene	Genetic Variant	Position	Transition	Consequence
*PNPLA3*	rs738409	chr22:43928847	C > G	Missense Variant
*PNPLA3*	rs2294918	chr22:43946236	A > G	Missense Variant
*GCKR*	rs780094	chr2:27518370	C > T	Intron variant
*MBOAT7*	rs641738	chr19:54173068	C > T	Downstream Transcript Variant
*TM6SF2*	rs58542926	chr19:19268740	C > T	Stop gained
*NCAN*	rs2228603	chr19:19219115	C > T	Missense Variant

## Data Availability

The corresponding author (B.S.R.) can provide the data supporting the study’s conclusions upon request. Because the data include information that would jeopardize the privacy of the patients who took part in the study, they are not made publicly available.

## Supplementary Materials

The following supporting information can be downloaded at: https://www.mdpi.com/article/10.3390/ijms26157409/s1.

## Abbreviations

HCC	Hepatocellular carcinoma
SNPs	Single nucleotide polymorphisms
PNPLA3	Patatin-like phospholipase domain-containing protein 3
GCKR	Glucokinase regulator
MBOAT7	Membrane-bound O-acyltransferase domain containing 7
NCAN	Neurocan
TM6SF2	Transmembrane 6 superfamily member 2
HBV	Hepatitis B virus
HBC	Hepatic C virus
ALD	excessive alcohol intake
AFB1	aflotoxin B1
NAFLD	Non-alcoholic fatty liver disease
NASH	Non-alcoholic steatohepatitis
T2DM	Type 2 diabetes
GWAS	Genome Wide Association Studies
BMI	Body mass index
BCLC	Barcelona Clinic Liver Cancer
MDR	Multifactor Dimensionality Reduction Software
VLDL	very low-density lipoproteins
ALT	alanine transaminase
RNA	Ribonucleic acid
DNA	deoxyribonucleic acid
HbsAg	Hepatic B surface antigen
MAF	Minor allele Frequency
CVC	Cross Validation Consistency
