# Supplementary material for: Contribution of PNPLA3, GCKR, MBOAT7, NCAN, and TM6SF2 Genetic Variants to Hepatocellular Carcinoma Development in Mexican Patients
Source: Int J Mol Sci. 2025 Aug 1;26(15):7409. doi: 10.3390/ijms26157409 (PMC12347070; doi:10.3390/ijms26157409)
Supplement: Supplementary file 1 [file ijms-26-07409-s001.zip › ijms-3453191-supplementary.pdf]

Supplementary Table S1. Association between GG genotype of *PNPLA3* rs738409 and subgroups of patients with HCC according to different etiologies, Child Pugh, and BCLC score.

	(n)	Case/control (%)	OR (CI 95%)	*P value*
Etiology	NAFLD (53)	55/31	2.68 (1.41-5.06)	0.003
	ALD (65)	55/31	2.68 (1.41-5.06)	0.003
	HCV (30)	54/31	2.68 (1.41-5.06)	0.003
Child-Pugh score	A (82)	58/31	3.07 (1.84-5.1)	<0.001
	B (62)	55/31	3.07 (1.84-5.1)	<0.001
	C (20)	54/31	3.07 (1.84-5.1)	<0.001
BCLC score	A (44)	55/31	2.68 (1.41-5.06)	0.003
	B (52)	55/31	2.68 (1.41-5.06)	0.003
	C (48)	50/31	2.68 (1.41-5.06)	0.003
	D (29)	55/31	2.68 (1.41-5.06)	0.003

NAFLD-Non alcoholic fatty liver disease, ALD-excessive alcohol intake, HCV, Hepatic C virus, BCLC, Barcelona Clinic Liver Cancer

Supplementary Table S2. Allelic frequencies among populations reported in 1000 Genomes Project Phase 3.

Gene	SNP	Allele	All	AFR	EUR	EAS	AMR	MEX*
PNPLA3	rs738409	G	26	12	23	35	48	55
		C	74	88	77	65	52	45
PNPLA3	rs2294918	G	79	90	63	82	79	83
		A	21	10	37	18	21	17
GCKR	rs780094	T	30	13	41	48	36	34
		C	70	87	59	52	64	66
MBOAT7	rs641738	T	37	32	44	22	34	30
		C	63	68	56	78	66	70
TM6SF2	rs58542926	T	7	2	7	9	6	6
		C	93	98	93	91	94	94
NCAN	rs2228603	C	96	99	93	94	98	98
		T	4	1	7	6	2	2

*Mexican ancestry in Los Angeles, CA.
